# Innate Lymphoid Cells in Intestinal Homeostasis and Inflammatory Bowel Disease

**DOI:** 10.3390/ijms22147618

**Published:** 2021-07-16

**Authors:** Angela Saez, Raquel Gomez-Bris, Beatriz Herrero-Fernandez, Claudia Mingorance, Cristina Rius, Jose M. Gonzalez-Granado

**Affiliations:** 1 LamImSys Lab, Instituto de Investigación Hospital 12 de Octubre (imas12), 28041 Madrid, Spain; angela.saez@ufv.es (A.S.); rgomez.imas12@correo.h12o.es (R.G.-B.); beatriz.herrero@uam.es (B.H.-F.); claumingo.imas12@h12o.es (C.M.); 2 Facultad de Ciencias Experimentales, Universidad Francisco de Vitoria (UFV), 28223 Madrid, Spain; 3 Departamento de Fisiología, Facultad de Medicina, Universidad Autónoma de Madrid (UAM), 28029 Madrid, Spain; 4 Faculty of Biomedical and Health Sciences, Universidad Europea de Madrid (UEM), Villaviciosa de Odón, 28670 Madrid, Spain; cristina.rius@universidadeuropea.es; 5 Centro Nacional de Investigaciones Cardiovasculares (CNIC), 28029 Madrid, Spain; 6 CIBER de Enfermedades Cardiovasculares, 28029 Madrid, Spain

**Keywords:** inflammatory bowel disease, innate lymphoid cells, intestinal homeostasis

## Abstract

Inflammatory bowel disease (IBD) is a heterogeneous state of chronic intestinal inflammation of unknown cause encompassing Crohn’s disease (CD) and ulcerative colitis (UC). IBD has been linked to genetic and environmental factors, microbiota dysbiosis, exacerbated innate and adaptive immunity and epithelial intestinal barrier dysfunction. IBD is classically associated with gut accumulation of proinflammatory Th1 and Th17 cells accompanied by insufficient Treg numbers and Tr1 immune suppression. Inflammatory T cells guide innate cells to perpetuate a constant hypersensitivity to microbial antigens, tissue injury and chronic intestinal inflammation. Recent studies of intestinal mucosal homeostasis and IBD suggest involvement of innate lymphoid cells (ILCs). These lymphoid-origin cells are innate counterparts of T cells but lack the antigen receptors expressed on B and T cells. ILCs play important roles in the first line of antimicrobial defense and contribute to organ development, tissue protection and regeneration, and mucosal homeostasis by maintaining the balance between antipathogen immunity and commensal tolerance. Intestinal homeostasis requires strict regulation of the quantity and activity of local ILC subpopulations. Recent studies demonstrated that changes to ILCs during IBD contribute to disease development. A better understanding of ILC behavior in gastrointestinal homeostasis and inflammation will provide valuable insights into new approaches to IBD treatment. This review summarizes recent research into ILCs in intestinal homeostasis and the latest advances in the understanding of the role of ILCs in IBD, with particular emphasis on the interaction between microbiota and ILC populations and functions.

## 1. Inflammatory Bowel Disease (IBD)

**Figure 1:**
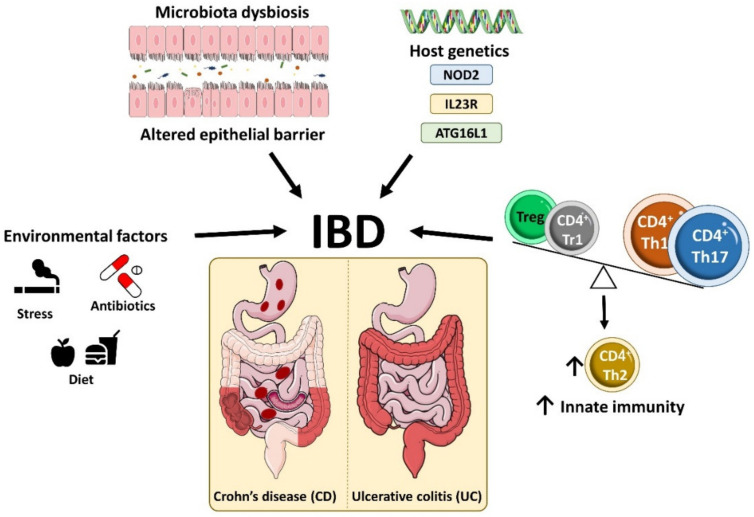
Etiology of inflammatory bowel disease. IBD is a heterogeneous state of chronic intestinal inflammation comprising two main clinical phenotypes, Crohn’s disease (CD) and ulcerative colitis (UC), which are distinguished by their symptoms, disease location and histopathological features. IBD arises from the interplay between environment factors, the gut microbiota and immunological factors in genetically susceptible individuals, which promotes intestinal barrier dysfunction, tissue damage and dysregulated innate and adaptive immune responses.

Inflammatory bowel disease (IBD) is a heterogeneous state of chronic intestinal inflammation comprising two main clinical phenotypes, Crohn’s disease (CD) and ulcerative colitis (UC), distinguished by symptoms, disease location and histopathological features [1]. The global incidence of IBD has increased considerably in recent years, with 6.8 million people affected globally in 2017 and an age-standardized prevalence of 84.3 (79.2–89.9) cases per 100,000 population [2]. IBD is characterized by chronic relapsing inflammation within the gastrointestinal tract with acute episodes and intervals of remission [3,4,5,6]. Shared clinical features of CD and UC patients include diarrhea, chronic abdominal pain and extraintestinal symptoms such as arthritis, oral ulcers, skin lesions and ophthalmological problems [1,7,8,9]. However, the two clinical manifestations of IBD show some differences in underlying pathology, inflammatory burden and prognosis [1,7,8,9]. UC is characterized by involvement of the colon, mainly in the mucosal layer, whereas CD patients have patchy lesions throughout the gastrointestinal tract, from the mouth to the anus, characterized by transmural inflammation, fistulae, abdominal abscesses and intestinal obstructions [8,9,10,11] (Figure 1).

## 2. Inflammatory Bowel Disease Etiology and Pathophysiology

IBD originates from an interplay between the environment, gut microbiota and immunological factors in genetically susceptible individuals, which promotes intestinal barrier dysfunction, tissue damage and dysregulated innate and adaptive immune responses [5,11,12] (Figure 1).

Numerous studies have demonstrated links between IBD and genetic factors. A family history of IBD is a risk factor for developing the disease [13,14], and IBD is also more prominent in monozygotic twin pairs than in dizygotic twin pairs [15]. Genome-wide association studies identified more than 200 IBD-associated genes, including genes related to host-mediated responses to the gut microbiota [16]. The first gene variants identified were of the muramyl dipeptide receptor NOD2 (nucleotide binding oligomerization domain containing 2) and were found to be associated with CD in Europeans and Americans [17,18]. However, this association was not found in China and Japan, revealing ethnic heterogeneity [19,20,21]. NOD2, expressed in innate immune cells, recognizes bacterial cell wall elements, and activates the nuclear factor kappa-light-chain-enhancer of activated B cells (NF-κB) signaling pathway. CD-susceptible NOD2 variants modify the recognition of bacterial wall components, increasing NF-κB activation in monocytes [22,23]. Other genes with variant forms showing strong and reproducible associations with CD are interleukin 23 Receptor (IL23R) gene and the autophagy-related gene ATG16L1 [24]. For example, some ATG16L1 polymorphisms in CD patients increase interleukin (IL-)1β production upon activation with NOD2 ligands [25]. This IL-1β potentiates intestinal inflammation by inducing T helper (Th)17 cell differentiation [26] (Figure 1).

**Figure 2:**
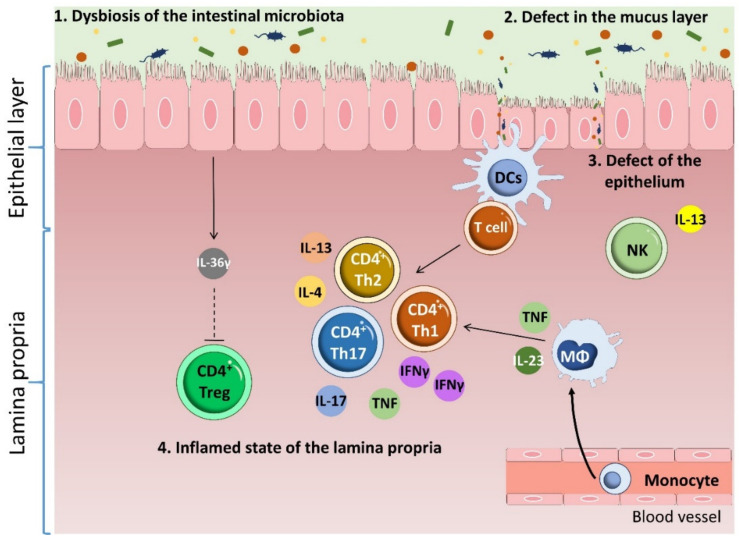
Pathophysiology of inflammatory bowel disease. Multiple factors contribute to IBD. Changes in the microbiota accompanied by thinning of the mucus layer induces a barrier breach that results in defects in the epithelium. Crossing of the barrier by microbiota components induces DC and macrophage activation, which induces infiltration of the intestinal tissue by inflammatory CD4 T cells. IBD patients have an increased content of proinflammatory Th1 and Th17 cells. This infiltration is accompanied by an increase in Th2 cell numbers and insufficient numbers of immune suppressing cells, such as Tregs. Inflammatory T cells guide the function of cells with an innate immune role, such as epithelial cells, fibroblasts and phagocytes, thus stimulating a constant hyperresponsiveness to microbial antigens and causing tissue injury and chronic intestinal inflammation.

A common feature of IBD in all patient categories is the infiltration of intestinal tissue by inflammatory CD4 T cells [27,28]. The predominant gut-infiltrating proinflammatory T cell populations in IBD are Th1 cells, which express the transcription factor T-bet and the cytokine interferon (IFN-)γ, and Th17 cells, which express the transcription factor retinoid (RAR)-related orphan receptor (ROR)γt and the cytokine IL-17 [29,30,31,32,33,34]. Infiltration by these populations is accompanied by an increase in the numbers of GATA-3-expressing IL-5- and IL-13-producing Th2 cells [35,36] and insufficient numbers of immune-suppressing cells, such as Foxp3-expressing T regulatory (Treg) and T regulatory type 1 (Tr1) cells [37,38,39] (Figure 2). Inflammatory T-cells guide the function of innate cells such as epithelial cells, fibroblasts and phagocytes, thus stimulating a constant hyperresponsiveness to microbial antigens [28] and causing tissue injury and chronic intestinal inflammation [40]. Some studies have reported IBD-associated dysbiosis, but it is still unclear if this is a cause or effect of IBD [41]. Chronic intestinal inflammation thus involves contributions from the intestinal microbiota, alterations to the epithelial intestinal barrier, the activation of immune cell, and reduced immune tolerance to bacteria [42].

## 3. Innate Lymphoid Cells (ILCs)

Recent discoveries have highlighted the role played in intestinal mucosal homeostasis and IBD by innate lymphoid cells (ILCs) [43,44,45,46,47,48]. ILCs play important roles in antimicrobial defense, and contribute to organ development, tissue protection and regeneration and mucosal homeostasis [49,50]. These cells belong to the innate immune response but originate from the same common lymphoid progenitor as lymphocytes [51,52]. Thus, while ILCs are the innate counterparts of T cells, they lack T-cell receptors generated through somatic recombination of antigen specific receptors. ILCs act early in the immune response by replying quickly to signals or cytokines produced by other cells. In contrast, naïve T cells require longer to act because they must first encounter antigens and undergo clonal proliferation to differentiate into functional and specific effector cells [51,53,54,55,56,57]. Other unique ILC characteristics that differentiate them from lymphocytes are that, unlike intestinal B, T, and natural killer (NK) cells, their intestinal population is not constantly replaced from the circulation [58] and that ILCs constantly produce their representative cytokines and other soluble molecules at steady state, contrasting with the on-demand production by T cells [48,59,60].

**Figure 3:**
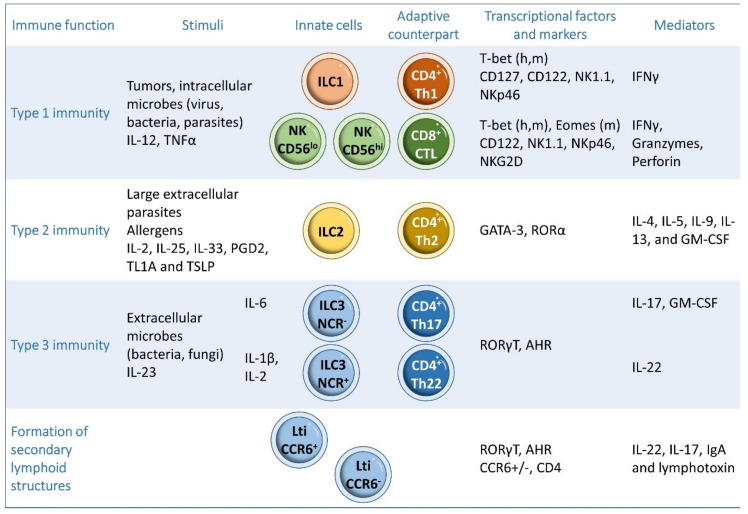
Classification of ILCs. ILCs can be classified into three subgroups: type 1 ILCs, including natural killer (NK) cells and ILC1s, type 2 ILCs (ILC2s) and type 3 ILCs (ILC3s). h: human, m: mouse.

ILCs can be classified into three subgroups according to their developmental pathways, specific key transcription factor, and cytokine expression [51,61,62,63]: type 1 ILCs (including natural killer (NK) cells and ILC1s), type 2 ILCs (ILC2s), and type 3 ILCs (including ILC3s and lymphoid tissue-inducers (LTis)) (Figure 3). ILC1s, ILC2s, and ILC3s, respectively, share functional similarities with CD4^+^ Th1, Th2 and Th17 cells, whereas NK cells have similar roles to CD8^+^ cytotoxic T cells. In addition, ILCs have many exclusive and pleiotropic functions [51,53,54,55,56,57] (Figure 3).

## 4. Type 1 ILCs

ILC1s are characterized by the expression of the α chain of the IL-7 receptor (CD127), whereas NK and ILC1s both express the IL-15 receptor β chain and the IL-2 receptor (CD122). NK cells and ILC1s also express NK1.1 (also known as killer cell lectin-like receptor subfamily B member 1C, Klrb1c) and natural cytotoxicity receptor NKp46 receptors. T-bet governs the function and differentiation of human and mouse ILC1s and NK cells, and mouse NK cells are also regulated by Eomes [64,65,66,67,68,69] (Figure 3).

**Figure 4:**
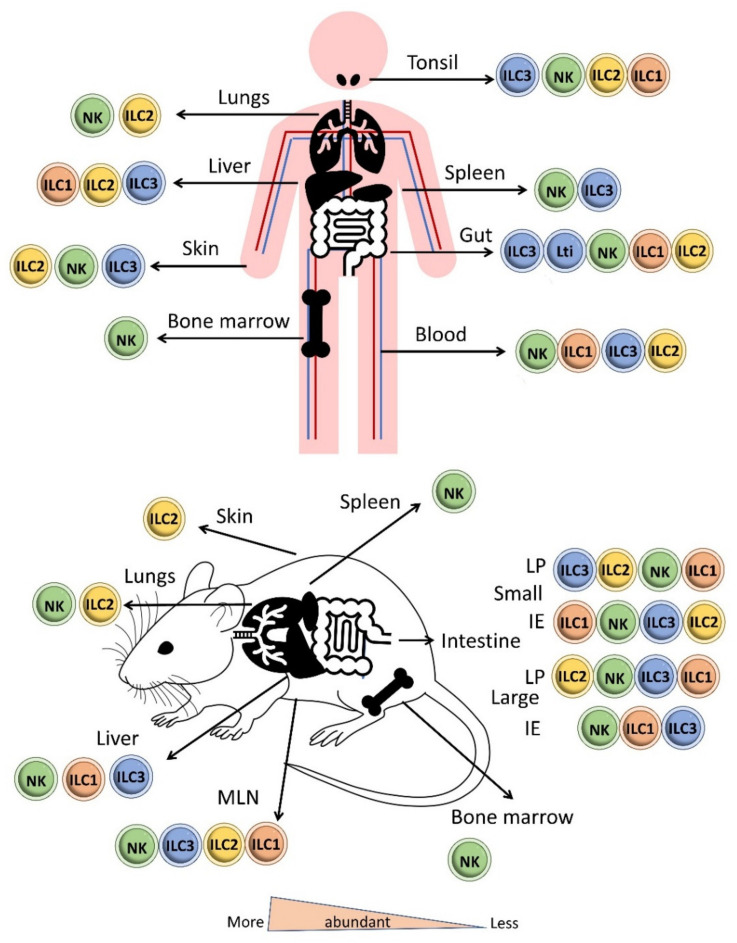
Distribution of ILCs in the body in human and mouse. NKs are circulating cells that are mainly found in the systemic circulation, cord blood, bone marrow, spleen, lungs and throughout the human gut. ILC1s are tissue-resident cells that mainly reside in intestinal tissues and tonsils but are also located in the liver, salivary glands, uterus and thymus. ILC2s are mainly found in adipose tissue, mesenteric lymph nodes, lungs, skin and tonsils. ILC3s are mainly present in mucosal tissue and at low levels in the spleen and liver. LTis cells are predominantly located in intestinal and lymphoid tissues, whereas NCR^+^ ILC3s and NCR^−^ ILC3s are more prominent in the skin and intestinal lamina propria. (LP: Lamina propria; IE: intraepithelial).

NK cells are circulating cells, broadly distributed in the blood and secondary lymphoid and peripheral organs. NK cells are mainly found in the systemic circulation, cord blood, bone marrow, spleen, lungs and throughout the human gut [70]. ILC1s are tissue-resident cells that predominantly reside in mucosal sites and are thus abundant in intestinal tissues and the tonsils [64,65,68,71]. ILC1s are also located in the liver, salivary glands, uterus and thymus [47,65,70,71,72,73] (Figure 4). NK cells, such as CD8^+^ T cells and ILC1s, like Th1 cells respond to intracellular pathogens such as viruses [74,75] and to tumors [76,77,78] (Figure 3).

NK cells distinguish target cells by stimulating and repressing the surface receptors NKp46, Natural killer group 2 member D (NKG2D), and in some mouse strains NK1.1. NK cells release IFN-γ and exert a cytotoxic effect in early defense against intracellular pathogens through the expression of cytotoxic compounds, the serine protease granzyme B and the pore-forming protein perforin, which causes apoptosis or osmotic cell lysis of the target cell [68]. NK cells also intervene in tumor immunosurveillance and in the balance of the immune system response by destroying some activated immune cells [79,80]. NK cells can be classified as CD56lo NKs and CD56hi NKs [81,82,83], with CD56lo NKs having a relatively more potent cytolytic activity and a lower capacity to sense and produce cytokines, especially IFN-γ [82,83].

ILC1s are broadly noncytotoxic but produce IFN-γ in response to IL-12 and tumor necrosis factor (TNF) to target intracellular pathogens such as viruses and bacteria [84,85,86,87,88].

## 5. Type 2 ILCs

ILC2s, the innate counterparts of Th2 cells, play important roles in the defense against large extracellular parasites by recruiting eosinophils and stimulating macrophages, granulocytes, mucus production by goblet cells and smooth muscle contraction [51,54]. ILC2s also intervene in allergen responses, promote airway inflammation and maintain and repair the airway epithelium [89,90,91,92]. ILC2s are also able to mount memory responses through a process termed trained immunity, generating efficient recall responses to reinfection [93]. These memory-like ILC2s are also important in response to allergens [94]. In fact, allergen-specific immunotherapy modifies the composition and heterogeneity of ILCs, among other innate immune cells, and brings them to the level found in healthy individuals. [95]

ILC2s produce IL-4, IL-5, IL-9, IL-13 and Granulocyte Macrophage Colony-Stimulating Factor (GM-CSF) upon activation by IL-2, IL-25, IL-33, prostaglandin (PGD)2, TNF-like ligand 1 A (TL1A), and Thymic Stromal Lymphopoietin (TSLP) [53,82,86,96,97,98,99,100]. ILC2 maintenance and function require the expression of the transcription factor GATA3 [101,102], and ILC2 function and differentiation also involves the transcription factors RORα, transcription factor T cell factor 1 (TCF1), BAF Chromatin Remodeling Complex (Bcl-)11b, ETS1, and Id2 [103,104] (Figure 3). ILC2s are mainly found in adipose tissue, spleen, mesenteric lymph nodes, lung, skin and tonsils [65,71] (Figure 4).

## 6. Type 3 ILCs

The generation and function of ILC3s depend on the expression of RORγt and aryl hydrocarbon receptor (AHR) [105,106]. ILC3s, like Th17s, promote immunity to extracellular bacteria such as Citrobacter rodentium and fungi and participate in tissue repair [50,51,107,108,109]. Type 3 ILCs include the LTi cells, natural cytotoxicity receptor (NCR)^−^ ILC3s, and NCR^+^ ILC3s. NCR is designated as NKp46 in mice and as NKp44 in humans [66,69,110,111].

During fetal development, LTis play an essential role in the generation of most secondary lymphoid organs, including the lymph nodes and Peyer’s patches, by teaching mesenchymal stromal cells to attract and retain hematopoietic cells [69]. LTis also stimulate stromal cells to recruit, retain and activate lymphocytes, as well as participating in the renewal and stimulation of epithelial cell defense and antiapoptotic programs [109,112]. LTis can be classified according to CCR6 expression [61,113]. C-C Motif Chemokine Receptor 6 (CCR6)^+^ ILC3s, including embryonic and mature LTi cells, produce IL-22, IL-17, immunoglobulin (Ig)A and lymphotoxin [61,114,115,116].

NCR^+^ ILC3s resemble Th22 cells, producing IL-22 upon stimulation with IL-1β, IL-23, and IL-2. In contrast, NCR^−^ ILC3s resemble Th17 cells in expressing IL-17 and GM-CSF in response to cytokines such as IL-6 [69,116,117,118,119,120]. NCR^+^ and NCR^−^ ILC3s are both able to produce IFN-γ [113].

## 7. ILCs and Intestinal Homeostasis

The gastrointestinal tract is a fundamental organ system for nutrient assimilation that is in constant contact with antigens from the diet, commensal bacteria and pathogens [39]. The intestinal immune system, therefore, needs to be permissive of dietary antigens and microbiota while effective against pathogens [39]. For this reason, the intestine is host to a multitude of organized cellular interactions in which ILCs play a fundamental role in regulating local immunity, inflammation and homeostasis [69]. Distinct ILC populations contribute to intestinal homeostasis in steady state conditions. Along the alimentary tract, the total number of ILCs increases from the oral cavity to the lower gastrointestinal tract, with the highest populations found in the ileum and colon [121].

**Figure 5:**
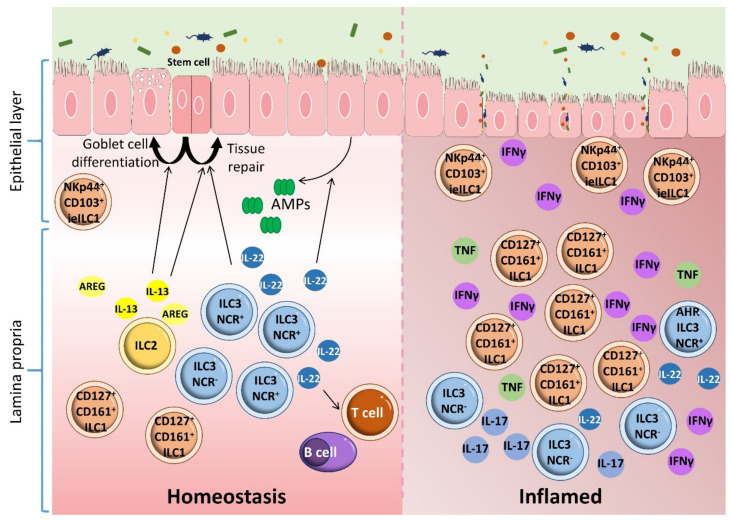
ILCs, homeostasis, and inflammatory bowel disease. There are two major subpopulations of ILC1s in the human gut: lamina propria ILC1s (CD161^+^ CD127^+^) and intraepithelial ILC1s (NKp44^+^ CD103^+^ CD127^−^). ILC2s are present in lower numbers than ILC1s and ILC3s. ILC2s contribute to epithelial barrier maintenance through the production of IL-13, which promotes the differentiation of intestinal epithelial stem cells toward turf cells and goblet cells. ILC2s also maintain intestinal epithelia homeostasis through the production of amphiregulin (AREG). ILC3s maintain gastrointestinal tract homeostasis by producing IL-22. This cytokine activates intestinal epithelial cells to produce antimicrobial peptides, enhances the renewal of epithelial cells, promotes tissue repair and modulates the homeostasis of adaptive immunity. Alteration of the ILC subset profile disturbs intestinal homeostasis and leads to inflammation in the gut. ILC3s and ILC1s are involved in the induction of inflammation and are closely related to IBD pathogenesis. This conclusion is supported by the amplification of intraepithelial IFN-γ-producing ILC1s in response to IL-12 and IL-15 in the gut of CD patients. This increase is accompanied by a reduction in NCR^+^ ILC3s, enhancing disease severity.

The human gut contains two major subpopulations of ILC1s: lamina propria ILC1s (CD161^+^, CD127^+^, NKp44^−^) [84] and intraepithelial ILC1s (NKp44^+^, CD103^+^, CD127^−^), which are comparable to classical NK cells in phenotype and cytotoxicity [122] (Figure 5). ILC2s are profuse in the fetal gut but are infrequent in the adult intestine, where they occur in lower number than ILC1s and ILC3s under steady state conditions [123]. ILC3s are mostly found in mucosal tissue and at low levels in the spleen, secondary lymphoid organs and the liver [65,70,71] (Figure 4). LTi cells are predominantly located in intestinal and lymphoid tissues, whereas NCR^+^ ILC3s and NCR^−^ ILC3s are more prominent in the skin and intestinal lamina propria [113,124]. ILC1s are the major fraction in the upper gastrointestinal tract, whereas ILC3s form the most numerous ILC populations in the ileum and colon [70].

ILCs, especially ILC3s, maintain gastrointestinal tract homeostasis through the following mechanisms: (1) sensing and interacting with the microbiota, (2) enhancing the renewal of epithelial cells and promoting tissue repair, (3) regulating the activation of intestinal epithelial cells to produce antimicrobial peptides and (4) modulating the homeostasis of adaptive immunity [46] (Figure 5).

**Figure 6:**
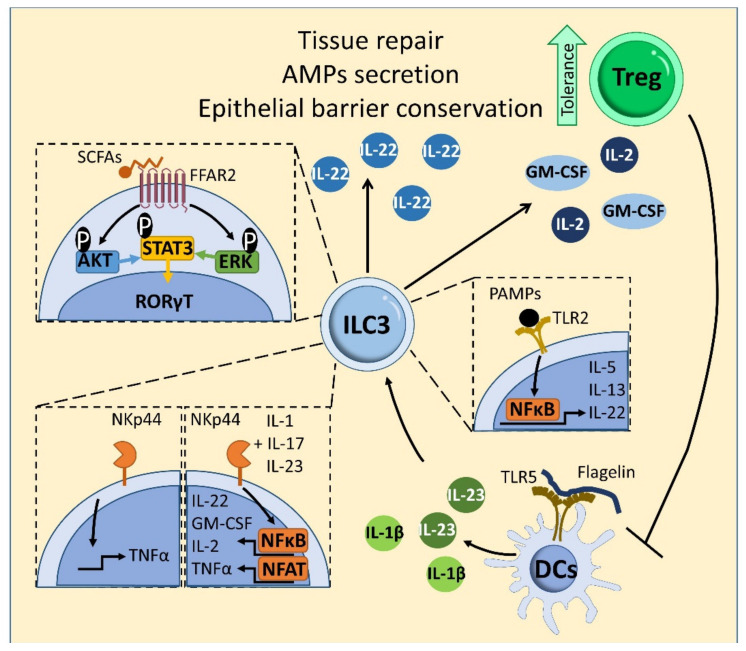
Microbiota-ILC3 interactions in intestinal homeostasis. Some SCFAs are recognized by the receptor FFAR2 on ILC3s, triggering IL-22 cytokine production by stimulating the AKT–STAT3 and ERK–STAT3 signaling pathways. TLR2 stimulation in human ILC3s promotes the production of IL-22, IL-13 and IL-5 upon activation of signaling via NF-κB and JAK (not shown). Stimulation of the TLR5receptor on dendritic cells (DCs) with the bacterial protein flagellin promotes the production of IL-23 and IL-1b, which induces production of IL-22, and IL-2 and GM-CSF by ILC3s, respectively. In human ILC3s, ligation of the natural cytotoxicity receptor NKp44 promotes the production of TNFα by ILC3s and, in combination with IL-1, IL-17 and IL-23, enhances ILC3 production of IL-22, GM-CSF, IL-2 and TNFα in a mechanism mediated by NF-κB and Nuclear factor of activated T-cells (NFAT) signaling. NKp44 receptor activation also increases the proportion of IL-22-producing ILC3s. IL-22 facilitates intestinal homeostasis by preservation of epithelial barrier function and promoting the secretion of antibacterial peptides (AMPs). IL-2 and GM-CSF contributes to intestinal tolerance mediated by Tregs. Tregs prevent ILC3-associated colitis by inhibiting IL-23- and IL-1β-induced IL-22 production.

**Figure 7:**
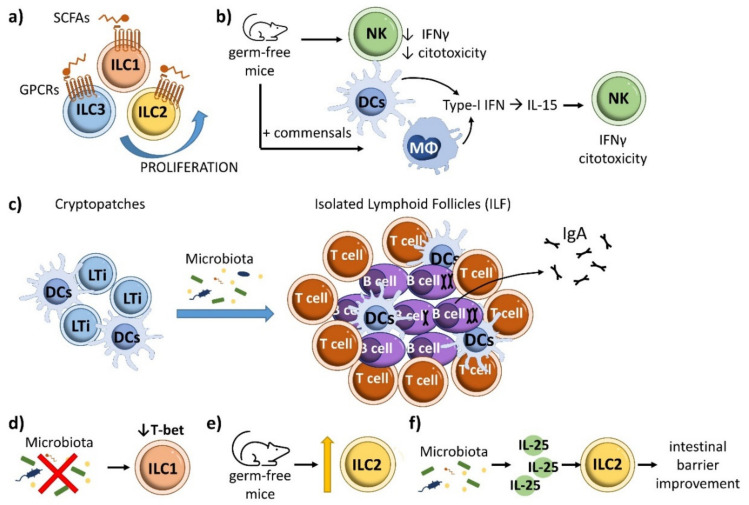
Influence of microbiota in other ILCs in intestinal homeostasis. (**a**) SCFAs produced by the commensal gut microbiota support optimal proliferation of ILC1, ILC2 and ILC3 populations by regulating G protein-coupled receptors (GPCRs). (**b**) In the absence of commensal bacteria, NK cells have reduced cytotoxicity and IFN-γ production. Colonization of germ-free mice with commensals increases NK cytotoxicity through the effect of dendritic cells and macrophage-derived type-I interferons on IL-15, which promotes NK cell terminal maturation. (**c**) LTi cells produce cryptopatches, which are transformed into isolated lymphoid follicles in a microbiota-dependent manner, supporting the production of intestinal IgA. (**d**) The gut microbiota enhances the expression of T-bet in ILCs. (**e**) The proportion of ILC2s in the gut is increased in the absence of commensal microbiota. (**f**) The microbiota regulates ILC2 function in the gut by promoting the release of IL-25, which drives ILC2s to improve intestinal barrier function.

The gut microbiota limits colonization by pathogens at barrier tissues through competition and the induction of immune responses. Commensals also favor immune homeostasis through the production of metabolites and their continuous collaboration with host cells [125]. The microbiota and its derived metabolites influence ILCs [126]. The microbiota directly influences ILC3 responses through sensing receptors [126,127,128]. Through the anerobic fermentation of undigested carbohydrates, the resident microbiota produces short-chain fatty acid (SCFA) end-products, including acetic acid, propionic acid, and butyric acid [129]. Some SCFAs induce ILC3 cell generation and IL-22 production through stimulation of the AKT–STAT3 and ERK–STAT3 signaling pathways [130] (Figure 6). Through their receptor free fatty acid receptor 2 (FFAR2), SCFAs also mediate host defense against Citrobacter rodentium and support tissue repair, as observed in a mouse model of dextran sodium sulfate (DSS)-induced intestinal injury [129,130]. IL-22 production by ILC3s is enhanced in germ-free or antibiotic-treated mice [131], and ILC3 generation and IL22 production is limited by addition of butyrate to ILC3s in vitro [132]. Moreover, dietary fiber-derived SCFAs produced by the commensal microbiota support optimal gut proliferation of ILCs, including ILC1, ILC2 and ILC3, by regulating G protein-coupled receptors (GPCRs) [44] (Figure 7a). In allergic asthma, butyrate, but not acetate or propionate, represses production of the type 2 cytokines IL-13 and IL-5 as well as proliferation and GATA3 expression in mouse and human ILC2s [133]. This inhibitory effect depends on an histone deacetylase (HDAC) suppressive effect rather than action through GPR41 or GPR43 [133], as also observed in ILC2-mediated lung inflammation induced by IL-33 and Alternaria [134]. Further experiments are needed to clarify the specific roles and mechanisms of SCFAs in ILC function.

ILC3s recognize microbial ligands through the engagement of NCRs such as NKp46, NKp44 and NKp30 [135]. In human ILC3s, binding of the natural cytotoxicity receptor NKp44 promotes the production of TNFα and, in combination with IL-1, IL-17 and IL-23 stimulation, enhances the production of IL-22, GM-CSF, IL-2 and TNFα through an NF-κB and nuclear factor of activated T-cells (NFAT)-dependent mechanism [136] (Figure 6). In addition, bacterial stimulation of NKp44 on ILC3s significantly enhances the proportion of IL-22-producing ILC3s [137].

ILC3s also express Toll-like receptors (TLRs) 1, 2, 5, 6, 7 and 9 (but not 3 and 4). These receptors recognizes pathogen-associated molecular patterns (PAMPs) derived from the gut microbiota [138]. In conjunction with IL-23 or IL-2, TLR2 stimulation in human ILC3s promotes the production of IL-22, IL-13 and IL-5 upon activation of NF-κB and Janus kinases (JAK) signaling [138] (Figure 6).

In addition to direct regulation, commensal bacteria control ILC3 activation indirectly by modulating myeloid cells and epithelial cells in the intestine [139,140]. The mouse intestinal microbiota promotes tissue-resident myeloid-cell production of IL-1β, which stimulates ILC3s to produce GM-CSF [139]. This GM-CSF, in turn, stimulates myeloid cells to promote Treg expansion and enhance tolerance [117,139] (Figure 6). Moreover, this microbiota-dependent myeloid-produced IL-1β triggers ILC3s to generate IL-2, which promotes Treg generation and intestinal tolerance to dietary antigens [140]. Tregs prevent ILC3-associated colitis by inhibiting IL-23 and IL-1β production from intestinal-resident C-X3-C Motif Chemokine Receptor 1 (CX3CR1)^+^ macrophages, which restrains ILC3-linked IL-22 production [141] (Figure 6). Additionally, CD11c^+^ myeloid dendritic cells produce IL-23 and IL-1β in response to enteric bacteria and contribute to ILC3 production of IL-22 [137]. Stimulation of dendritic-cell TLR5 with the bacterial protein flagellin promotes the generation of IL-23, which drives IL-22 production by ILC3s (Figure 6).

NK development does not require microbiota; however, NK cell function is altered in the absence of commensal bacteria, evidenced by reduced cytotoxicity and IFN-γ production in germ-free or antibiotic-treated mice [68]. In line with this finding, NK cytotoxicity is increased by colonization of germ-free mice with commensals [142], an effect mediated by the actions of dendritic cells and macrophage-derived type-I interferon on IL-15 [143,144]. IL-15 promotes terminal NK cell maturation, thus the commensal microbiota indirectly controls the generation of NK cells [126,145] (Figure 7b). The microbiota also mediates the postnatal transformation of LTi-cell cryptopatches into isolated lymphoid follicles, which support the production of intestinal IgA [146] (Figure 7c). The impact of the microbiota on ILC1 development and function is not well established, except that the absence of microbiota reduces ILC expression of T-bet [113,147] (Figure 7d).

ILC2s are the least abundant intestinal ILCs [70]. Germ-free and specific-pathogen-free (SPF) mice show no differences in the number and frequency of ILC2s or the expression of ILC2 markers (the receptors for IL-25 and IL-33, IL-7Rα, T1/ST2, and c-Kit) [148]. This would suggest that the microbiota is not required for ILC2 development [126]; however, lack of commensal microbiota increases the percentage of ILC2s in the gut [149] (Figure 7e). Microbiota-ILC interactions contribute to the maintenance of a tight and healthy epithelial intestinal barrier. Human ILC2s directly recognize bacteria through the expression of TLR1, TLR4 and TLR6 [150]. Other studies show that the microbiota regulates ILC2 function in the gut by promoting the release of IL-25, which drives ILC2-mediated improvement of the intestinal barrier [131,151] (Figure 7f). The cytokine IL-33 stimulates ILC2s and reduces Clostridium difficile colitis [152], whereas selective genetic abrogation of T-bet in ILCs promotes ILC2 expansion and increases ILC2 activity, resulting in protection against Trichinella spiralis infection and inflammatory colitis [153].

ILC2s also contribute to gut homeostasis and the progression of inflammation through other mechanisms. These include conservation of the epithelial barrier through the production of IL-13, which promotes the differentiation of intestinal epithelial stem cells toward turf and goblet cells. Turf cell differentiation enhances epithelial sensing, whereas goblet cells increase mucus production [151]. ILC2-derived IL-13 favors self-renewal of intestinal epithelial stem cells [154]. ILC2s are stimulated by IL-33, IL-25 and thymic stromal lymphopoietin, as well as eicosane cytokines such as prostaglandin D2 (PGD2) and leukotriene D4 [155]. ILC2s also maintain epithelial homeostasis through the IL-33-dependent production of the growth factor amphiregulin (AREG), which binds to epithelium-expressed epidermal growth factor receptor (EGFR) [156]. ILC2-expressed IL-13 promotes cup-cell hyperplasia, modifying the intestinal epithelial barrier and promoting acidophilic cell proliferation by stimulating the production of IL-5 and IL-9 [157]. In contrast, T-bet-producing ILCs like ILC1s increase intestinal barrier permeability through the action of IFN-γ and TNF-α [158,159].

ILC3s also contribute to intestinal homeostasis by mechanisms independent of microbiota interaction. ILC3-derived IL-22 has been linked to the induction of several factors that promote intestinal homeostasis and preserve the epithelial the barrier function, such as tight and gap junction proteins, mucins and cytokine receptors [160]. Moreover, reduced gut IL-22 production impairs microbiota symbiosis [109,161]. IL-22, mostly derived from ILC3s, also stimulates gut epithelial cells to secrete antibacterial peptides that promote long-term tolerance to diet-derived antigens [162]. This action is mediated by the activation of IL-22 receptors expressed on nonhematopoietic intestinal cells, primarily intestinal epithelial cells [163]. IL-22 stimulation of Paneth cells promotes production of the antimicrobial proteins Reg3b, Reg3y, S100A8 and S100A9 [164].Moreover, ILC3-derived IL-22 increases the proliferation of Lgr5^+^ intestinal epithelial stem cells [165]. ILC3-derived IL-22 and lymphotoxin induce fucosyl transferase (Fut2) expression in epithelial cells, favoring the fucosylation of intestinal epithelial cells [166]. Epithelial fucose is used as a dietary carbohydrate by commensal bacteria, establishing a mechanism of host-microbiota symbiosis that contributes to intestinal homeostasis [167,168]. Through their intrinsic expression of major histocompatibility complex class II (MHC-II), MHC-II^+^ CCR6^+^ ILC3s contribute to the maintenance of intestinal immune tolerance by promoting the apoptosis and deletion of CD4^+^ T cells specifically targeting commensal bacteria in the lamina propria [169,170].

## 8. ILCs and Inflammatory Bowel Disease

IBD is associated with intestinal alterations and dysbiosis [171,172]. Together with other immune cell populations, ILCs participate in IBD pathogenesis through interaction with the microbiota, modulation of epithelial barrier integrity, and the production of cytokines such as IL-22 and IL-17 [43,45,47,57,118,173,174] (Figure 5). Aberrant alterations to the ILC population balance disturb intestinal homeostasis, leading to gut inflammation. IBD and gut inflammation are closely linked to the balance between ILC1s and ILC3s (Figure 5). For example, the intraepithelial IFN-γ-producing ILC1 population is expanded in CD patients in response to IL-12 and IL-15 [84,122], and a similar alteration is seen in anti-CD40-induced colitis in Recombination activating gene (Rag1)^−/−^ mice [122]. This increase is accompanied by a reduction in NCR^+^ ILC3s, increasing disease severity [84,122,175]. The ILC population in the lamina propria of inflamed tissues from CD patients is skewed toward the CD127^+^ ILC1 subset [84]. These CD127^+^ ILC1s produce high amounts of IFN-γ in response to IL-12 and IL-18 [83].

Not all ILC3 populations are reduced in IBD. ILCs from CD patients have augmented gene expression of some ILC3s cytokines, transcription factors and cytokine receptors such as IL-12A and IL-22, RORC and AHR, and IL23R [5,176]. IL-23 induces ILC3 expression of IL-17 and IL-22 [49,111,112]. Experiments in mouse colitis models have linked IL-23-responsive NCR^−^ ILC3s to the development IBD through the production of IL-17A, IL-22, and IFN-γ [177,178]. IL-23–reactive ILC3s have also been detected in human mucosa-associated lymphoid tissue, including intestinal Peyer’s patches and tonsils [50]. Unlike ulcerative colitis patients, CD patients have elevated expression of IL-17A and IL-17F in mucosal ILCs [176]. In an anti-CD4 and anti-CD90 antibody-depleting mouse colitis model, IL-6 was found to contribute to the activation and production of IL-17A, IL-22 and IFN-γ in intestinal ILC3s, a finding confirmed in isolated lamina propria cells from IBD patients [119]. Intestinal ILCs, including ILC3s, enter and exit cryptopatches in a highly dynamic process. During colitis, ILC3s mobilize from cryptopatches and initiate inflammatory immune cascades that result in intestinal inflammation [120].

In homeostasis, ILC3-derived IL-22 binds to the IL-22R to trigger the production and maintenance of IL-18 mRNA and promote IL-18 signaling in epithelial cells. In the mouse, IL-22 promotes IL-18 signaling in epithelial cells, and impaired IL-18 strengthens intestinal inflammation, harming the intestinal barrier and producing mucosal inflammation [179]. CD patient mucosa contains a reduced number of IL-22-producing NCR^+^ ILC3s [84], and the subsequent reduction in IL-22 may compromise barrier integrity and contribute to IBD development [180]. IBD patients also have reduced levels of regenerating islet-derived protein (Reg)IIIγ, RegIIIb, Fut2 and mucin protein Muc2 [162,166], in addition to reduced expression of the tight junction protein claudin-2 and impaired regeneration of intestinal epithelial cells [119,181]. ILC3s express high levels of the receptor for TL1A, death receptor 3 (DR3). DR3 antibody ligation exacerbates colitis by triggering ILC3 production of GM-CSF via stimulation of the p38 MAPK pathway, resulting in the accumulation of CD11b^+^ CD11c^+^ myeloid cells and facilitating the loss of ILC3s from the intestine via an IL-23-dependent mechanism [182]. Consequently, an antibody-mediated DR3 blockade ameliorates colitis, possibly by repressing the harmful effects of ILC3s [182]. In summary, NCR^+^ ILC3s, important for maintaining intestinal homeostasis by producing IL-22, are reduced in IBD, whereas IL-17-producing NCR^−^ ILC3s contribute to disease development.

Like ILC1s, gut NK cells suppress infection but enhance inflammation and IBD [79,83,183,184]. Spontaneous intestinal inflammation is related to high endoplasmic reticulum stress in intestinal epithelial cells. NK cells promote endoplasmic reticulum stress in intestinal epithelial cells by increasing expression of NKG2D and worsen intestinal inflammation by recognizing and killing stressed intestinal cells [185]. NK cell numbers are increased in the lamina propria of the colon of CD patients and promote CD by facilitating CD4^+^ T cell proliferation and Th17 differentiation [186,187]. The intestinal mucosa of CD patients has a disrupted balance between NKp44^+^ and NKp46^+^ NK cells [188]. NKp46^+^ NK cells may mediate CD by producing IFN-γ, which activates intestinal inflammatory macrophages in CD-patient intestinal mucosa [188]. Primary sclerosing cholangitis, an idiopathic chronic hepatobiliary-system disorder linked to UC, is associated with increased numbers of IFN-γ-producing ILC1s [189].

Less is known about the role in IBD played by ILC2s. Treatment with IL-33 or transfer of ILC2s ameliorates mouse intestinal inflammation through an AREG-dependent mechanism [156]. However, IL-33 is increased in the intestinal mucosa of IBD patients, enhancing colitis [190,191]. Moreover, ILC2 numbers are higher in inflamed regions of UC-patient intestine than in noninflamed regions or the intestines of healthy individuals [175]. IL-13^+^ ILC2 numbers are increased in a mouse model of oxazolone-induced colitis [192], suggesting that ILC2s may have a proinflammatory action in UC.

ILC2 activation is evident in CD patient blood from the elevated levels of the IBD markers SLAMF1 (signaling lymphocytic activation molecule family member 1) and human leukocyte antigens (HLA-)DR [16,193], the latter related to the capacity of ILCs to mediate antigen presentation [190]. In CD patients with active disease, SLAMF1^+^ ILC2 frequency correlates negatively with disease severity and SLAMF1^+^ ILC2s appear to represent a mature population that produces IL-13 and high levels of prostaglandin D2 receptor 2 (PTGDR2/CRTH2), CD161, and GATA3 [194].

ILC2s participate in IBD through their ability to sense microbiota, be stimulated by cytokines and maintain the intestinal barrier. The early IL-33–dependent expansion of ILC2s in CD is driven by activation of the intracellular pattern recognition receptor NOD2, as evidenced in gut-derived ILC2s from CD patients [195] and SAMP1/YitFc (SAMP) mice, a mouse strain that spontaneously develops a progressive, chronic intestinal inflammation similar to CD (reviewed in [196]). NOD2 detects muramyl dipeptide, a peptidoglycan by-product found in cell walls of both gram-positive and gram-negative bacteria. ILC2s also regulates important functions during early immune responses through the expression of C-C motif chemokine receptor 8 (CCR8), the receptor for CC chemokine ligand 1 (CCL1)/T cell activation specific gene 3 (TCA3) [197,198]. CCL1-CCR8 signaling is a key mediator of monocyte and lymphocyte chemoattraction and is implicated in Th2 and Treg-mediated inflammatory diseases [199,200,201,202,203]. CCR8 signaling protects mice against acute intestinal damage through the action of a subgroup of IFNγ-producing ILCs in a DSS mouse model of colitis; however, it remains to be determined whether these cells are proper ILC2s, ILC1s or Tbet^+^ exILC3s [204].

ILC2s contribute to the maintenance of intestinal barrier integrity through indirect mechanisms. ILC2s express the surface inhibitory receptor killer-cell lectin like receptor G1 (KLRG1), which promotes ILC2 activity by activating GATA-3 [205]. In a DSS mouse model of colitis, KLRG1 was inhibited as a result of increased miR-21a-5p content in M1-macrophage–derived exosomes; the resulting decrease in E-cadherin expression weakened intestinal barrier integrity [206] and aggravated IBD. In addition, ILC2s release IL-13 in response to IL-25 released from Tuft cells activated by berberine, an isoquinoline alkaloid extracted from Coptis chinensis that acts on the bitter taste receptor TAS2R. The ILC2-derived IL-13 triggers the differentiation of intestinal stem cells into Turf and goblet cells, which repair injured intestine [207]. While these studies go some way to clarifying the role of ILC2s in IBD, much further work is needed to fully define how ILC2s contribute to immune impairment and protection in IBD.

**Figure 8:**
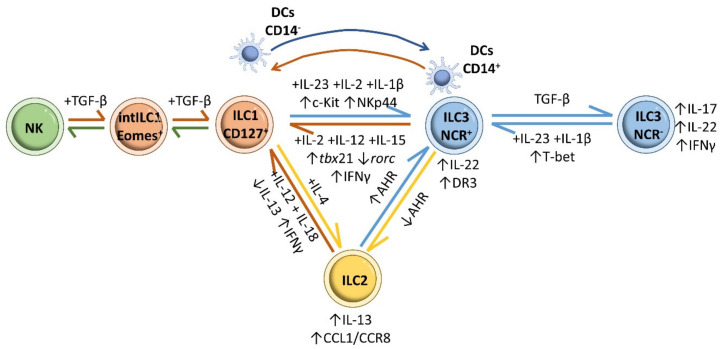
ILC plasticity. Prolonged exposure of ILC3s to the type 1 polarizing cytokines IL-2, IL-12 and IL-15 induces their conversion to CD127^+^ ILC1 cells, with downregulation of RORC, upregulation of TBX21 and IFN-γ production. Reverse differentiation of ILC1s to ILC3s is driven by IL-23, IL-2 and IL-1β. Moreover, CD14^−^ DCs mediate the transformation of ILC1s into ILC3s in vivo by promoting synthesis of c-kit and NKp44 in ILC1s, whereas CD14^+^ DCs are implicated in the transformation of NCR^+^ ILC3s into ILC1s. IL-23 and IL-1β stimulation induces the conversion of NCR^−^ ILC3s into the more proinflammatory NCR^+^ ILC3 subset through T-bet upregulation. The reverse differentiation from NCR^+^ ILC3s to NCR^−^ ILC3s is promoted by high levels of TGF-β. Removal of AHR enriches the effect of intestinal ILC2s, whereas increased AHR expression restrains ILC2 function while increasing ILC3 function. Treatment with IL-12 and IL-18 reduces IL-13 expression in ILC2s, shifting them to an IL-13^−^ IFN-γ^+^ ILC1 phenotype. ILC2-derived ILC1s can revert to ILC2s in response to IL-4. NK cells (CD49a^−^ CD49b^+^ Eomes^+^) differentiate into intermediate type 1 innate lymphoid (intILC1, CD49a^+^ CD49b^+^ Eomes^+^) populations and ILC1s (CD49a^+^ CD49b^−^ Eomes^int^) in response to cytokine and TGF-β signaling.

The altered ILC population profile in IBD is a reflection of ILCs plasticity (Figure 8). In the inflamed intestinal mucosa of CD patients, the numbers of CD127^+^ ILC1 is increased at the cost of a decline in ILC3s [208]. This plasticity appears to be linked to downregulation of RORC, upregulation of TBX21 and the production of IFN-γ upon prolonged exposure to the type 1 polarizing cytokines IL-2, IL-12 and IL-15 [69,84,209]. ILC3 conversion to the ILC1 phenotype is reversible, and differentiation of ILC1s to ILC3s is driven by IL-23, IL-2, IL-1β, and retinoic acid [84,208]. ILC1-to-ILC3 differentiation involves IL-23-mediated activation of STAT3 in CD117^−^ NKp44^−^ type1 ILCs, and this action was linked to the positive correlation found between STAT3 rs744166 risky allele “A” and disease severity in a cohort of CD patients [210]. STAT3-induced modulation of ILC3 IL-22 production also protects the mouse intestine from infection [211,212]. Interconversion between ILC3s and ILC1s also involves a contribution from dendritic cells. CD14^−^ DCs mediate the transformation of ILC1s into ILC3s in vivo by promoting the expression of c-kit and NKp44, whereas CD14^+^ DCs are implicated in the transformation of NCR^+^ ILC3s into ILC1s [208].

IL-23 is also implicated in the conversion of NCR^−^ ILC3s into the more proinflammatory NCR^+^ ILC3 subset via T-bet upregulation during Typhoid bacillus infection in mice [84,113]. IL-23^−^ and IL-1β-induced transformation of NCR^−^ ILC3s into NCR^+^ ILC3s has also been reported in vitro in cells isolated from human tonsils and fetal gut [84], and in cells isolated from the skin of psoriasis patients [213]. NCR^−^ ILC3-to-NCR^+^ ILC3 differentiation appears to depend on sustained Notch signaling [214,215]. The reverse transformation, from NCR^+^ ILC3s to NCR^−^ ILC3s, is promoted by high levels of TGF-β, countering the effects of Notch [215]. An active equilibrium between NCR^+^ ILC3s and NCR^−^ ILC3s is essential for ensuring a balance between antibacterial immunity and homeostasis maintenance in the intestine; disruption of this balance promotes IBD [45,216].

In an inflammatory environment, ILC2s can convert to some or all of the typical ILC1 and ILC3 phenotypes [157]. The frequency of IL-13-producing ILC2s is increased in the intestinal mucosa of CD patients, and in vitro experiments show that IL-12 and IL-18 promote the conversion of ILC2s into ILC1s [47,217,218]. Likewise, removal of AHR enriches the effect of intestinal ILC2s and potentiates anti-helminth immunity, whereas AHR activation restrains ILC2 function while increasing ILC3 action, thus supporting antibacterial immunity [219]. It is likely that ILC2–ILC3 interchange triggered by changes in intestinal AHR expression impairs the intestinal immune response in IBD [45]. As with ILC3s and ILC1s, interconversion between ILC2s and ILC1s is bidirectional: ILC2-derived ILC1s can revert to ILC2s in response to IL-4 [220,221].

Plasticity has also been reported in NK-ILC1s in a tumor microenvironment. NK cells (CD49a^−^ CD49b^+^ Eomes^+^) differentiate into intermediate type 1 innate lymphoid cells (intILC1, CD49a^+^ CD49b^+^ Eomes^+^) and ILC1 (CD49a^+^ CD49b^−^ Eomes^int^) cells in response to cytokine and TGF-β-signaling [222,223].

In summary, in steady state conditions, ILC3s constitute the major intestinal ILC population, but during active inflammation, IFN-γ–producing ILC1s and IL-17–producing ILC3s increase, while IL-22–producing ILC3s decrease. This redistribution is linked to increased disease severity.

## 9. Conclusions

The intestinal epithelium has to facilitate nutrient absorption while at the same time impeding the entry of pathogens and toxins. In addition, the gut needs to support a symbiotic interaction with commensal bacteria that promotes their proabsorption activity while keeping them in the intestinal lumen. The intestinal immune system is thus in contact with antigens from food, pathogens and commensal bacterial. The ability to discern between helpful and injurious signals, and to maintain intestinal homeostasis, depends on highly controlled, multicellular processes. ILC subpopulations are important mediators of intestinal homeostasis and play protective and pr-inflammatory roles in IBD. ILCs exert their protective function by controlling the microbial niche and by regulating epithelial barrier maintenance and repair. In contrast, ILCs cause inflammation by releasing cytokines and modulating the innate and adaptive immune responses. ILCs directly and indirectly sense the microbiota, which influences the ILC-mediated immune response; gut bacteria and ILCs modify each other bidirectionally. Alterations to the relative proportions of the different ILC subpopulations are linked to ILC interpopulation transformations and determine the origin, maintenance or prevention of disease. The development of new treatments for IBD will require detailed knowledge of the mechanisms that determine changes to ILC subpopulation frequencies and function, and how these populations mediate their protective and inflammatory effects.

## Abbreviations

AHR	Aryl hydrocarbon receptor
CCL1	C-C chemokine ligand 1
CCR8	C-C motif chemokine receptor 8
CD	Crohn’s disease
IBD	Inflammatory bowel disease
ILCs	Innate lymphoid cells
EGFR	Epidermal growth factor receptor
Fut2	Fucosyl transferase 2
GPCRs	G protein-coupled receptors
NCR	Natural cytotoxicity receptor
NK	Natural killer
NOD2	Nucleotide binding oligomerization domain containing 2
LTis	Lymphoid tissue-inducers
PAMPs	Pathogen-associated molecular patterns
PGD2	Prostaglandin D2
PTGDR2	Prostaglandin D2 receptor 2
RORγt	RAR-related orphan receptor gamma t
SCFAs	Short-chain fatty acids
SLAMF1	Signaling lymphocytic activation molecule family member 1
SPF	Specific-pathogen-free
Th	T-helper
TL1A	TNF-like ligand 1 A
TLRs	Toll-like receptors
Treg	T regulatory
Tr1	T regulatory type 1
UC	Ulcerative colitis
